# Delineating the Diagnostic Concordance Between Pediatric Lower Urinary Symptoms Scoring and Voiding Diary in Pediatric Lower Urinary Tract Dysfunction

**DOI:** 10.7759/cureus.42463

**Published:** 2023-07-25

**Authors:** Necmi Bayraktar, Serdar Tekgul

**Affiliations:** 1 Urology Department, Dr. Burhan Nalbantoglu State Hospital, Nicosia, CYP; 2 Pediatric Urology, Hacettepe University School of Medicne, Ankara, TUR

**Keywords:** voiding dysfunction, urinary incontinence, urinary tract infections (utis), diagnostic tools, voiding diary (vd), pediatric lower urinary tract symptoms scoring (plutss), lower urinary tract dysfunction (lutd)

## Abstract

Background: This retrospective research endeavored to conduct a comparative evaluation of the Pediatric Lower Urinary Tract Symptoms Scoring (PLUTSS) system and the Voiding Diary (VD). The correlation between these diagnostic tools, their prognostic value for treatment outcomes in pediatric Lower Urinary Tract Dysfunction (LUTD), and their relationship with patients' sociodemographic characteristics were also explored.

Methodology: The study data for the cohort established between December 2005 and September 2006 were obtained from a specialized thesis, while the subsequent expansion from 2022 to 2023 involved a prospective approach, including an additional 73 patients, resulting in a total of 113 pediatric patients (79 females and 34 males). Comprehensive diagnostic evaluations, such as urinalysis, urine culture, renal function tests, urinary tract ultrasound, uroflowmetry-electromyography (EMG), and post-voiding residual urine measurement (PVR), were conducted. The patient's symptoms were assessed using the Pediatric Lower Urinary Tract Symptom Score (PLUTSS) and a two-day-three-night voiding diary.

Results: The correlation between the PLUTSS and VD was not absolute but substantial concerning daytime frequency and incontinence. Notably, PLUTSS emerged as the primary predictor of treatment outcomes. No significant association was discerned between sociodemographic characteristics, such as socioeconomic status, sibling count, toilet training, school performance, patient personality, and LUTD diagnosis or prognosis.

Conclusion: The findings underscore the prognostic value of PLUTSS for treatment outcomes in pediatric LUTD. Although a significant correlation was observed between PLUTSS and VD, they are not interchangeable. As a result, concurrent utilization of both tools is endorsed for comprehensive diagnosis, follow-up, and treatment planning in pediatric LUTD.

## Introduction

Lower Urinary Tract Dysfunction (LUTD) is a widely recognized health issue among the pediatric population, particularly characterized by an involuntary contraction of the urethral sphincter during urination [1]. Manifesting in a myriad of urinary complications such as urinary tract infections (UTIs) and urinary incontinence, the complexity and prevalence of LUTD underscore its importance as a public health concern [2-5].

Determining precise prevalence estimates for pediatric LUTD is inherently complicated, primarily owing to the diversity in diagnostic criteria and methodologies employed across different clinical contexts. Nevertheless, estimates suggest that up to 30% of children could be impacted by LUTD, thereby spotlighting the pressing need for effective diagnostic and management strategies from both an individual and public health perspective [2,5,6].

The clinical presentation of pediatric LUTD is often marked by recurrent UTIs and urinary incontinence, both of which can have profound impacts on a child's physical health and psychosocial well-being. Further complications, such as the risk of repeated UTIs leading to potential kidney damage due to vesicourethral reflux (VUR), escalate the concern in the clinical management of affected children [7-9].

In this clinical landscape, the value of accurate, reliable, and non-invasive diagnostic tools is paramount. Instruments such as the Voiding Diary (VD) and the Pediatric Lower Urinary Tract Scoring System (PLUTSS) have gained wide acceptance due to their proven efficacy in symptom evaluation and monitoring of treatment response [2,10,11]. Complementing these tools, non-invasive techniques like uroflowmetry-electromyography (EMG) and sonographic post-voiding residual volume measurement (PVR) offer valuable insights into the physiological aspects of voiding, thereby enhancing the precision of diagnosis and targeted treatment planning [12,13]. In cases where LUTD presents more complex or severe symptoms, invasive methodologies such as urodynamic measurements have proven to be of substantial clinical value [7,14].

Despite the considerable strides made in pediatric LUTD research, key challenges persist. Central among these is the ongoing debate regarding the optimal diagnostic tools and treatment strategies, fueled by a lack of consensus and the shortage of comparative studies scrutinizing tools such as VD and PLUTSS. This discord leaves pertinent questions unresolved, particularly concerning the interchangeability of these tools [5].

This study is positioned to address this gap by examining the compatibility and interchangeability of VD and PLUTSS as diagnostic tools for pediatric LUTD. Through this comprehensive analysis, we aim to augment the understanding of LUTD and contribute to the development of more evidence-based treatment plans and patient care management strategies, thereby addressing existing knowledge gaps in pediatric healthcare and enriching the body of research in this field.

## Materials and methods

Data collection

Our study, conducted from December 2005 through September 2006, was based on data derived from thesis research. This study involved a carefully selected cohort of 40 patients. Informed consent was obtained from all participants or their respective guardians, ensuring ethical compliance. This cohort served as the initial basis for our study. Subsequently, from October 2022 to May 2023, we expanded the cohort by including an additional 73 patients, resulting in a total of 113 pediatric patients (79 females and 34 males).

Eligibility criteria

The inclusion criteria targeted children presenting with LUTS, who were not previously diagnosed with or treated for Lower Urinary Tract Dysfunction (LUTD). The exclusion criteria, designed to be stringent, were set to exclude children with systemic diseases, those with a preexisting diagnosis of neurogenic bladder, or those currently receiving treatment for LUTD. This strategy ensured a homogeneous and specific sample size for our investigation.

Study design and sampling technique

After inclusion, the participants underwent a systematic and detailed assessment process. The initial data collection included a comprehensive history of each patient, supplemented by an exhaustive physical examination. Further investigations incorporated routine urinalysis and urine cultures to rule out tract infections, a potential confounder. Uroflowmetry-EMG, a potent tool for diagnosing urinary disorders, and Post-Void Residual (PVR) urine volume were measured using ultrasonography. To ensure holistic data collection, all participating families were instructed to complete the PLUTSS questionnaire during their initial visit (Table 1).

**Table 1:** Pediatric lower urinary tract symptoms score (PLUTSS, English version) Note: Turkish version of PLUTSS was used in the study.

Questions	Answer options								
1. Does your child have urinary incontinence (peeing while not on the toilet) during the day?	No	Sometimes			1-2 times/day			3 or more times/day	
	0	1			3			5	
2. If Yes to Question 1	A few drops		Only underwear wet				Outer clothing layers wet		
	1		3				5		
3. Does your child have urinary incontinence (peeing while not on the toilet) during the night?	No		1-2 nights/week			3-5 nights/week			6-7 nights/week
	0		1			3			5
4. If Yes to Question 3	Underwear or pajamas wet					Bed wet			
	1					4			
5. My child goes to the toilet to pee …	Less than 7 times/day					7 or more times/day			
	0					1			
6. My child has to strain to pee.	No					Yes			
	0					3			
7. My child experiences pain when s/he pees.	No					Yes			
	0					1			
8. My child pees intermittently when on the toilet.	No					Yes			
	0					2			
9. My child has to go to revisit the toilet to pee soon after s/he pees.	No					Yes			
	0					2			
10. My child has to run to the toilet when s/he feels the need to pee.	No					Yes			
	0					1			
11. My child can hold his/her pee by crossing his/her legs, squatting, or doing the “pee dance.’’	No					Yes			
	0					2			
12. My child wets his/her clothes before reaching the toilet.	No					Yes			
	0					2			
13. My child does not pass stool every day.	No					Yes			
	0					2			
Quality of Life									
If your child experiences any of the symptoms/issues mentioned above, does this affect his/her family or social life?	Not at all			Sometimes				Seriously affects	
	0			1				5	

Additionally, they received detailed instructions for maintaining the Voiding Diary (VD) over a period of two days and three nights, commencing from Friday Night. Following the initial evaluations and data collection, we initiated a tailored treatment plan for each participant. After a six-week treatment period, follow-up assessments were conducted to evaluate the effectiveness of the intervention.

Data analysis

Statistical analyses were conducted using non-parametric tests due to the non-normal distribution of the data. The analyses were performed using SPSS software, version 26.0 (IBM Corp., Armonk, NY), and the Shapiro-Wilk test was utilized to validate the non-parametric nature of our dataset. We applied the Mann-Whitney U test and the Wilcoxon signed-rank test for various comparisons. The former was employed to compare the medians between two independent groups, while the latter facilitated pairwise comparisons within the same group for pre-and post-treatment assessments. To evaluate the agreement level between the Pediatric Lower Urinary Tract Symptom Score (PLUTSS) and the Voiding Diary (VD), we calculated the kappa coefficients in compliance with Cohen's interpretation guidelines. Values ≤ 0 indicated no agreement, 0.01-0.20 implied none to slight agreement, 0.21-0.40 signified fair agreement, 0.41-0.60 denoted moderate agreement, 0.61-0.80 represented substantial agreement, and 0.81-1.00 reflected almost perfect agreement. We further executed linear correlation and bivariate regression analyses to identify variables significantly associated with treatment outcomes. In this context, Fisher's Freeman-Halton test was applied to discern the relationship between pre-treatment uroflowmetry flow curves and initial PLUTSS scores. The statistical significance was established at a p-value of less than 0.05.

Ethical considerations

This study adhered to the guidelines outlined in the Declaration of Helsinki and was approved by the Northern Cyprus Ministry of Health’s Ethics Committee (Issue: Project Code: 35/23).

## Results

Our present scholarly endeavor involved an examination of a diverse cohort comprising 113 pediatric patients, including 79 females and 34 males, with an average age of 7.6 years. Remarkably, the plurality of patients, specifically 89.3%, were embedded within a conventional family structure. Normal infancy was documented in an impressive 96% of the instances, and the successful completion of toilet training within the conventionally accepted age bracket of 18-36 months was affirmed in 83% of the cohort. Notably, a minor subset of patients (6.2%) encountered developmental delays during infancy. Furthermore, the birth modalities manifested a bifurcation into 69% vaginal and 31% cesarean deliveries.

A thorough personality assessment unveiled a timorous temperament in 36.5% of the subjects, an extroverted predisposition in 54.5%, and introverted tendencies in a mere 9%. Comorbidities associated with voiding dysfunction encompassed urinary tract infection (43%), VUR (14.5%), incontinence (83%), and constipation (36%). Pertinently, the primary grievances at the time of initial admission predominantly encompassed daytime and nighttime incontinence, as well as urinary tract infections, as corroborated by 86% of the patient pool.

Employing the Fisher's Freeman-Halton test, optimized for our limited patient cohort size, yielded a statistically significant correlation between pre-treatment uroflowmetry flow patterns and the initial Pediatric Lower Urinary Tract Symptom Score (PLUTSS). Specifically, a propitious trend between an elevated PLUTSS score and the likelihood of aberrations in uroflowmetry flow curves was evident (p<0.05). Yet, a noteworthy absence of any substantial linkage between pre-treatment uroflowmetry results and the rationale for the first clinical presentation was observed (p>0.05).

An application of the Wilcoxon and Mann-Whitney U tests to dissect pre- and post-treatment uroflowmetry curves elicited a significant deviation (p<0.05). Table 2 furnishes an encapsulation of the kappa coefficients adopted to quantify the concordance and agreement between the VD and PLUTSS.

**Table 2:** Kappa coefficient assessment of agreement across key indicators between VD and PLUTSS VD: Voiding Diary; PLUTSS: Pediatric Lower Urinary Tract Symptom Score

	Cohen’s Kappa(Ϗ)	Agreement
Daytime incontinence	0.76	Good
Daytime incontinence severity	0.47	Moderate
Nighttime incontinence	0.38	Poor
Nighttime incontinence severity	0.46	Moderate
Daytime Frequency	0.69	Good

The implementation of the Wilcoxon test to juxtapose pre-and post-treatment VD and PLUTSS data, specifically pertaining to daytime incontinence, did not yield any significant discrepancy (p>0.05). Nevertheless, a significant divergence in the severity of daytime incontinence between pre-and post-treatment phases was discerned (p<0.05), with a propensity for pre-treatment diary entries to overestimate severity.

Upon a meticulous evaluation of the quantitative PLUTSS scores, a significant decrement, amounting to 38%, was discernible in the post-treatment phase (p<0.05). A bivariate correlation analysis, conducted to evaluate treatment outcomes, demarcated the pre-treatment PLUTSS score (-44%) as the sole significant determinant (p<0.01). A conspicuous absence of a statistically robust correlation between pre-treatment uroflowmetry flow curves, admission grievances, and fluctuations in PLUTSS scores was detected (p>0.05). However, an inverse correlation surfaced between the baseline PLUTSS score and the therapeutic outcome. A higher inaugural PLUTSS score was associated with diminished therapeutic efficacy (Table 3).

**Table 3:** Correlation analysis among ΔPLUTSS score, initial uroflowmetry, clinical presentation, and pre-treatment PLUTSS score ΔPLUTSS Score: Represents the difference in the Pediatric Lower Urinary Tract Symptom Score (PLUTSS) pre- and post-treatment. Initial Uroflowmetry: Indicates the initial uroflowmetric findings recorded prior to the commencement of treatment. Clinical Presentation: Represents the primary clinical complaints or symptoms reported at the time of patient admission. Pre-treatment PLUTSS Score: Represents the Pediatric Lower Urinary Tract Symptom Score (PLUTSS) recorded for the patient before the initiation of treatment. Pearson Correlation: The Pearson Correlation coefficient signifies the strength and direction of a linear relationship between two variables. The coefficient ranges between -1 and 1, with -1 indicating a perfect negative correlation, +1 indicating a perfect positive correlation, and 0 indicating no correlation. p-value: Denotes the statistical significance. A p-value less than 0.05 indicates statistical significance. In this context, only the correlation between ΔPLUTSS Score and Pre-treatment PLUTSS Score is statistically significant (p=0.012), suggesting that the ΔPLUTSS Score value is sensitive to changes in the Pre-treatment PLUTSS Score. N/A: Stands for Not Applicable. This is used in cells where it is not logically relevant to describe correlations and p-values, such as the correlation between the same variables. "*": Indicates results that are statistically significant. Here, the negative correlation between ΔPLUTSS Score and Pre-treatment PLUTSS Score is statistically significant (p<0.05).

	ΔPLUTSS Score Correlation	p-value (ΔPLUTSS)	Initial Uroflowmetry Correlation	p-value (Uroflowmetry)	Clinical Presentation Correlation	p-value (Presentation)	Pre-treatment PLUTSS Score Correlation	p-value(Pre-treatment PLUTSS)
ΔPLUTSS Score	1	N/A	-0.180	0.242	0.117	0.466	-0.378*	0.012
Initial Uroflowmetry	-0.180	0.242	1	N/A	0.083	0.643	0.209	0.183
Clinical Presentation	0.117	0.466	0.083	0.643	1	N/A	-0.066	0.657
Pre-treatment PLUTSS Score	-0.378*	0.012	0.209	0.183	-0.066	0.657	1	N/A

Finally, the implementation of a Linear regression model to unravel potential influences on PLUTSS score variations revealed the baseline total PLUTSS score as an independent determinant, oblivious to the pre-treatment uroflowmetry curve and the presence of UTI. Notably, pre-treatment PLUTSS scores exerted a considerable impact on therapeutic outcomes (p=0.027) (Table 4).

**Table 4:** Influence of pre-treatment factors on the difference in PLUTSS scores (a linear regression analysis) Independent Variables: These are the factors being evaluated for their impact on the difference in Pediatric Lower Urinary Tract Symptom Score (PLUTSS). Unstandardized Coefficients (B): These represent the raw coefficients as computed directly from the data. Standard Error (SE): This measures the standard deviation of the sampling distribution of a statistic, most commonly of the mean. Standardized Coefficients (Beta): These are the coefficients obtained when the independent and dependent variables are standardized before the analysis. T-Value: The T-value measures the size of the difference relative to the variation in the data. Significance (p-value): The p-value represents the likelihood that the observed effect occurred by chance if the null hypothesis is true. A p-value less than 0.05 is generally considered statistically significant. Here, the correlation between Pre-treatment PLUTSS Score and the PLUTSS Score difference is statistically significant (*p<0.05), suggesting that Pre-treatment PLUTSS Score has a significant impact on the PLUTSS Score difference.

Independent Variables	Unstandardized Coefficients (B)	Standard Error (SE)	Standardized Coefficients (Beta)	T-Value	Significance (p-value)
Initial Uroflowmetry	-1.365	1.181	-0.107	-0.689	0.496
Clinical Presentation	1.705	2.551	0.095	0.624	0.431
Pre-treatment PLUTSS Score	-1.021	0.557	-0.454	-2.332	0.027*

## Discussion

Lower Urinary Tract Dysfunction (LUTD) is a multifaceted pathology that complicates the path to an accurate diagnosis and an optimal treatment plan, often hindering these processes if relying solely on patient history [1]. Echoing our collected data and clinical experience, we found that the subjective nature of scoring systems such as the Pediatric Lower Urinary Tract Symptoms Scoring (PLUTSS) combined with the inherent challenges of maintaining detailed Voiding Diary (VD) records underscore the necessity of utilizing non-invasive diagnostic tools. On the other hand, underscores the value of initial PLUTSS scores in predicting treatment outcomes for pediatric patients grappling with LUTD.

Urinary tract infections (UTIs) are common in children with bladder and bowel issues, occurring in approximately 45% of children with LUTS. Nearly 30% of these children also experience constipation. Therefore, it is important to carefully examine the association between UTIs and their relationship with constipation in children with LUTS. Both PLUTSS and VD methods can help assess LUTS and constipation. However, our two-day VD may not be sufficient to accurately record constipation in some children. Extending the diary could help with this but could also make it more difficult to manage. In our group of patients, a high number (85%) had incontinence, indicating that UTIs and incontinence are common in children with LUTD. Additionally, constipation, which affects almost 30% of patients, needs to be carefully investigated and treated. Furthermore, Machado et al. found a link between LUTS and hard-to-treat constipation by carefully studying the time it takes for food to move through the colon (colonic transit time) [15]. Our results are seen to be compatible with the current literature.

The VD covers only a short period compared to the PLUTSS, which could explain why they do not always agree. Interestingly, they do not always agree on the severity of daytime urinary incontinence, which can be subjective. This makes it difficult to distinguish between minor and severe wetting conditions. More objective tests, such as the pad test, could provide more accurate results; however, we need to consider how these could affect a child's motivation and mental well-being.

When it comes to nighttime incontinence, the PLUTSS and VD might disagree because they cover different periods and use different observation methods. While the PLUTSS provides an estimated assessment over seven nights, VD provides a more precise assessment over three nights. However, VD might not always detect incontinence in children who wet the bed less often or occasionally. Therefore, the International Children's Continence Society (ICCS) standardization paper suggests using both questionnaires and voiding diaries to evaluate and monitor the treatment of LUTS [1,3].

In terms of how often a child needs to urinate during the day, the PLUTSS and VD generally agree, indicating that they are reliable for measuring this. Previous research has shown that the PLUTSS can be useful for diagnosing LUTD without the need to perform invasive tests and to check how well a treatment is working [7]. Hooman et al. tried to improve the reliability of the diagnosis by using the Child Behavior Checklist (CBCL) and bladder volume wall index (BVWI) in children with LUTS [12]. While the CBCL and BVWI did not help with diagnosis, the CBCL was helpful for identifying children with behavioral issues and bedwetting, and the PLUTSS was the best predictor of LUTS [16].

Changes in PLUTSS scores are easy to follow over time, providing a simple way to track treatment progression. In our study, the main differences in the PLUTSS scores before and after treatment were mainly due to the initial PLUTSS score, regardless of other factors. This makes the initial PLUTSS score a key indicator of treatment effectiveness.

Study limitations

Our study had a few limitations that warrant attention. Primarily, our small patient cohort size may limit the broader applicability of our findings. Furthermore, the short observation timeframe of VD could have led to the under-representation of LUTS.

The subjective nature of our measures, the PLUTS scoring and VD, may introduce bias. The absence of a control group potentially limited our ability to account for natural progression or spontaneous improvement. Our study didn't fully account for all LUTS-related factors like psychological aspects, and the follow-up duration may not have been enough to assess long-term treatment outcomes. Also, our reliance on patient or caregiver adherence for recording symptoms could have added variability.

For future research, larger sample sizes, longer observation periods, a control group, comprehensive inclusion of variables, and strategies for improving adherence to diagnostic tools should be considered to overcome these limitations.

## Conclusions

In conclusion, PLUTSS was the sole predictor of treatment outcomes. A negative correlation existed between PLUTSS scores and treatment outcomes. No correlation was observed between PLUTSS and VD, indicating they should not be substituted. It is suggested that using both tools together may be a better diagnostic and follow-up approach. Further research with larger patient groups and diverse demographics is required to confirm this.
